# EOR-1/PLZF promotes WAH-1/AIF-dependent compartment-specific corpse clearance

**DOI:** 10.1038/s41420-025-02874-2

**Published:** 2025-11-28

**Authors:** Nathan Rather, Aladin Elkhalil, Melvin Williams, Karen Juanez, Rashna Sharmin, Ginger Clark, Shai Shaham, Piya Ghose

**Affiliations:** 1 The University of Texas at Arlington, Arlington, TX USA; 2 The Rockefeller University, New York, NY USA

**Keywords:** Cell biology, Developmental biology

## Abstract

Programmed cell death (PCD) is a crucial, evolutionarily conserved process required for development and homeostasis. We previously described a genetically non-canonical apoptotic, highly ordered cell death program called Compartmentalized Cell Elimination (CCE) in the *C. elegans* morphologically complex tail-spike epithelial cell (TSC). Here, we define a role for the transcription factor EOR-1/PLZF as an important, compartment-specific, regulator of CCE. We identify EOR-1 specifically in the dying cell’s own clearance, which can function downstream of CED-3/caspase, which is essential for TSC killing. Whereas prior studies implicate EOR-1 in programmed cell killing, we provide mechanistic detail in a new developmental cell elimination context. Specifically, we find EOR-1/PLZF positively regulates Apoptosis Inducing Factor homolog WAH-1 during CCE. We identify WAH-1 as a new contributor to two steps of soma-specific clearance during CCE, acting in the dying cell: corpse recognition-internalization, and phagosome maturation-corpse resolution following engulfment. In the absence of EOR-1/PLZF as well as WAH-1/AIF, the TSC soma remains uninternalized, persisting with exaggerated nuclei previously undescribed. We suggest a role of the scramblase SCRM-1 in TSC soma internalization by the phagocyte and show spatiotemporal specificity of the presentation of the canonical apoptotic corpse recognition signal phosphatidylserine (PS) on the TSC. We also report that in the absence of CPS-6/Endonuclease G, the internalized TSC soma corpse arrests at the phagolysosomal stage, also yielding exaggerated nuclei. This suggests that WAH-1 also promotes DNA degradation during CCE during phagosome maturation. Our work provides new molecular and cell biological insights into CCE and expands our understanding of the transcriptional regulation of corpse clearance.

## Introduction

Programmed cell death (PCD) is a genetically-encoded and evolutionarily-conserved cell elimination process vital for normal development and homeostasis [1], with defects linked to disease [2–4]. Apoptosis, the most well-studied form of PCD, has certain morphological hallmarks such as cell shrinkage, chromatin condensation, nuclear fragmentation, and mitochondrial fragmentation [1, 5, 6]. The genetic control of apoptosis is conserved and includes caspase proteases and their regulators, including the pro-apoptotic Apaf-1, the anti-apoptotic Bcl-2, and the pro-apoptotic BH3-only in mammals [7–9]. Forms of non-apoptotic, caspase-independent regulated cell death have also been described, such as Linker Cell-type Death (LCD) [10].

Following cell killing, cell remains must be efficiently removed via phagocytosis to avoid secondary necrosis and autoimmune consequences [11, 12]. The steps and mechanism of apoptotic corpse phagocytosis have been well described [13, 14]. Dying cells are recognized [15] and engulfed by the phagocyte [16] to become fully internalized and encapsulated in a phagocytic vesicle called a phagosome. This corpse-containing phagosome undergoes a series of maturation steps that entail progressively increased acidification [17] and introduction of lysosomal hydrolases [18] that lead to the ultimate resolution of the corpse. These corpse-resolving events are regulated by conserved pathways [19, 20].

Despite in-depth studies on apoptotic cell death and clearance, gaps in our knowledge remain with regard to cell elimination mechanisms across other cell death programs and diverse cell types. Are the clearance mechanisms of other forms of PCD the same as those of apoptosis? Another poorly understood aspect of cell clearance is whether this occurs in the same way for the removal of distinct cell compartments for specialized cells. For example, neurons have complex morphologies containing a cell body, processes/projections-axons, and dendrites. These types of cells have regions that differ vastly in subcellular architecture and their surrounding microenvironment. The killing/dismantling of these regions and their ultimate phagocytosis would presumably occur via different mechanisms. However, this intriguing point is not well-studied.

Much of what we know about the molecular basis of apoptotic cell killing and clearance has been gleaned using the powerful genetic model organism, the nematode *C. elegans* [21–24]. Newer studies have described the role of engulfment machinery genes in other contexts, such as region-specific neuronal sculpting and function [25] as well as polar body removal [26], and in cell clearance following non-apoptotic LCD [27, 28].

We previously described the tripartite cell killing program Compartmentalized Cell Elimination (CCE) of the polarized tail-spike epithelial scaffolding cell (TSC) in the *C. elegans* embryo [29]. Briefly, CCE entails three distinct and stereotyped cell elimination events across different cell compartments—namely, the soma and the proximal and distal segments of the single posteriorly-directed process. The first visible indication of CCE is a membrane “nicking and severing” event at the junction between the soma and proximal segment of the process. The binucleate soma then rounds, superficially resembling an apoptotic morphology [29]. The single process of the TSC segments into two morphologically distinct sub-compartments that exhibit degenerative morphologies resembling two different forms of neurite pruning [29]. Notably, as the CCE program occurs 10 times slower than most apoptotic nematode cells, it allows for detailed examination of cell elimination events at the gross and subcellular level [14, 29, 30].

Molecularly, while CCE is caspase-dependent, it is genetically distinct from apoptosis [31–33]. While CCE does require the main *C. elegans* caspase CED-3, CED-3 here is not regulated by the BH3-only homolog EGL-1 [33]. This has led to the identification of several non-canonical regulators of CED-3 and hence cell killing [31–33]. In addition to killing, CCE has also served as a fertile ground for the discovery of genes regulating compartment-specific cell clearance. Interestingly, during engulfment, the canonical engulfment machinery operates only on the soma corpse of the TSC and not for the TSC process [29]. Clearance of the TSC process, specifically the distal remnant, requires the cell-cell fusogen EFF-1 in the phagocyte for phagosome sealing, a generally poorly understood step of phagocytosis [29]. While the internalization mechanisms are distinct, during CCE, the canonical phagosome maturation machinery is employed to resolve the different TSC compartments once internalized by the phagocyte [29].

In the present study, we identify the conserved master transcription factor EOR-1/PLZF as a previously undescribed regulator of compartment-specific (soma) cell clearance that operates in the dying TSC. While EOR-1 has been implicated in cell killing/death specification for both apoptosis [34] and non-apoptotic LCD [35], we show here a role for EOR-1 in the TSC further downstream of caspase function in cell elimination. Rather than overall cell killing, in the context of CCE, EOR-1 supports TSC soma clearance specifically, promoting its internalization and resolution, therefore playing a role in compartment-specific cell removal. We present EOR-1 as a positive transcriptional regulator of the Apoptosis inducing factor WAH-1/AIF and show that WAH-1 functions within the TSC to promote both the presentation of the phosphatidylserine (PS) “eat-me” signal and for DNA degradation. Finally, we report unexpected cell biology of the dying cell in the form of region and temporally-disparate presentations of PS and previously unreported cell corpse morphology.

## Results

### Compartmentalized Cell Elimination has stage and region-specific morphological hallmarks and is regulated via a non-canonical apoptotic pathway

**Fig. 1:**
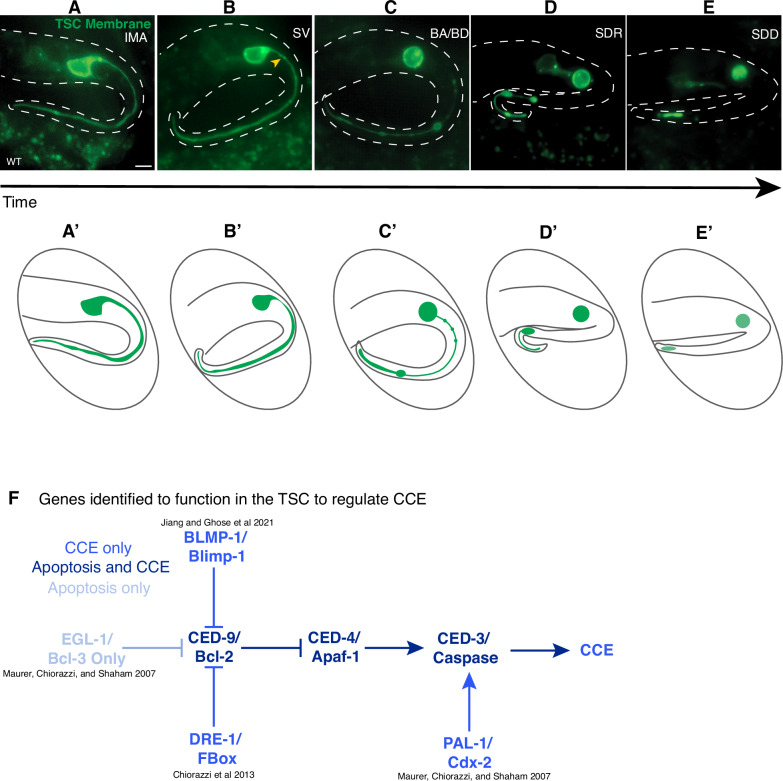
Compartmentalized cell elimination has stage and region-specific morphological hallmarks and is regulated via a noncanonical apoptotic pathway. **A–E’** Stages of CCE with cartoon representations. **A**, **A’** IMA, intact mature cell *n* = 10; **B**, **B’** SV, Membrane severing stage, yellow arrowhead, soma-process junction to be severed *n* = 5; **C**, **C’** BA, beading attached; BD, beading detached *n* = 10; **D**, **D’** SDR, soma-distal retracted *n* = 6; **E**, **E’** SDD, soma, distal degrading. *n* = 10 biologically independent animals each with similar results. **F** Genes identified as regulators of tail-spike cell (TSC) death.

We have previously shown that the TSC dies through an elaborate program with distinct elimination morphologies across different compartments observed at different stages [29]. The TSC is a morphologically complex cell that extends a single posteriorly-directed process when intact (Fig. 1A, A**’**) (IMA, intact, mature) that serves to scaffold the animal’s tail-tip. At CCE onset, a membrane nicking-severing event is observed at the junction between the soma and process (Fig. 1B, B**’**). In the “beading” stages (BA/BD) (Fig. 1C, C**’**), the binucleate soma rounds in a manner superficially resembling apoptotic cell death and the process shows beading proximal to the soma and retraction distally into a newly formed distal node. The proximal segment of the process is cleared away first and the distal process fully retracts into this distal node (Fig. 1D, D**’**) (soma, distal retraction, SDR) leaving behind a soma corpse and a distal remnant (soma, distal degrading, SDD) (Fig. 1E, E**’**). The remnants of the soma and distal process are subsequently phagocytosed stochastically before the animal hatches. Notably, the TSC soma and process are phagocytosed by different neighboring phagocytes and involve different molecular machinery [29].

CCE is a rich setting to describe new genetic regulation of programmed cell elimination. CCE is dependent on the main *C. elegans* caspase CED-3 [29, 33], with the TSC remaining intact into the larval stage in *ced-3* mutants. This death program is, however, genetically distinct from apoptosis (Fig. 1F) [33] as it does not require EGL-1/BH3-only for death initiation [33]. Several new regulators have been found to control TSC killing and the onset of CCE [33]. PAL-1/Cdx-2 transcription factor directly binds to *ced-3* regulatory sequences to promote its transcription during CCE [33]. The F-box protein DRE-1 helps degrade CED-9/Bcl2 [31], and the BLMP-1/BLIMP-1 transcription factor also represses CED-9 transcription in order to promote CCE [32]. Notably, these studies that focus on genes that act upstream of CED-3/caspase all involve one CCE defect, namely, the failure in killing, such that the TSC remains fully intact in mutants for these genes.

Conversely, in our other work, we have shown that clearance of the soma and process compartments require distinct molecular machineries [29]. While engulfment of the TSC soma requires the canonical apoptotic engulfment pathways, clearance of the TSC process does not [29]. This presents the TSC as a powerful model to study compartment-specificity of cell clearance. With this in mind, we performed an unbiased forward genetic screen for CCE defects of the TSC using a membrane marker strain for the TSC (TSC-myrGFP) [29]. This screen revealed that the TSC process, but not the soma, requires the fusogen EFF-1 for phagosome sealing [29], with only the TSC process persisting in mutants. The present study stems from another TSC screen mutant that displays a persisting soma only, rather than process or a fully intact cell, and highlights the distinct mechanisms required for the elimination of cell compartments.

### The transcription factor EOR-1/PLZF promotes CCE compartment-specifically and can function downstream of CED-3 caspase

**Fig. 2:**
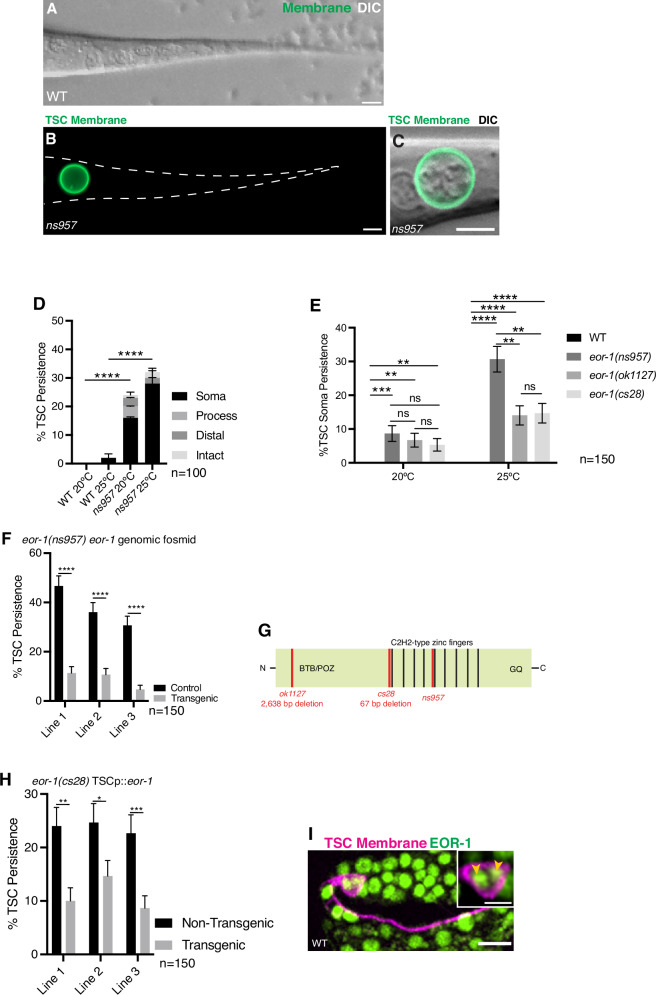
The transcription factor EOR-1/PLZF promotes CCE compartment-specifically. **A** Wild-type control for L1 TSC defects showing lack TSC at L1 (GFP and DIC, lack of GFP signal due to absence of TSC). **B** Mutant *ns957* at L1 stage showing persisting TSC soma, *n* = 10 biologically independent animals, each with similar results. **C** Enlarged view of (**B**) *ns957* persisting soma to show DIC view. Green, TSC membrane. **D** Quantification of *ns957* CCE defects **E** Quantification of CCE defects of other *eor-1* alleles. **F** Quantification of *eor-1* fosmid DNA rescue of *ns95*7. **G** Gene structure of *eor-1* showing mutation sites. **H** Rescue of *eor-1(cs28)* mutant CCE defect with TSC promoter-driven *eor-1*. **I** *eor-1* expression is shown in a wild-type embryo at the IMA stage of CCE. Magenta, TSC membrane. Green, *eor-1* promoter-driven EOR-1 translational fusion. Yellow arrowheads, nuclei. *n* = 10 biologically independent animals each with similar results. Scale bar, 5 μm except for inset of (**I**) which is 2.5 μm. Data are mean ± s.e.m. Statistics: two-tailed unpaired student’s *t* test, see Supplementary Table 3 for individual *P*-values.

From our forward genetic screen, we obtained a mutant, *ns957*, which, unlike wild-type (Fig. 2A), bears an inappropriately persisting and enlarged cell body only, but with no process (Fig. 2B, C). This CCE defect increased when animals were raised and scored at 25 °C when compared to 20 °C (Fig. 2D). We examined the morphology of the *ns957* mutant more carefully using Differential Interference Contrast (DIC) microscopy. Notably, unlike apoptotic corpses [36], we observed in our *ns957* mutant enlarged nuclei and a non-refractile cytoplasm (Fig. 2C).

We next aimed to identify the relevant gene mutated in *ns957*. Following Whole Genome Sequencing, genomic/fosmid DNA rescue (Fig. 2F), and examination of other null alleles (Fig. 2E [37–40]), we found that our screen mutant has a causative lesion in the gene encoding the transcription factor EOR-1/PLZF, resulting in an E570K change in the Zn finger domain (Fig. 2G). We also found from cell-specific rescue experiments that *eor-1* acts cell-autonomously in the TSC (Fig. 2H), and that EOR-1 is localized to the TSC nuclei consistent with the notion that it acts as a transcription factor (Fig. 2I). We tested whether EOR-1 functions in CCE together with its previously-identified transcriptional partners [40, 41], namely EOR-2, the MAU-2 cohesin loader, and the SWI/SWNF chromatin remodeling complex member SWSN-1, and found a persisting soma defect in these mutants similar to *eor-1(cs28)* (Supplementary Fig. S1A–G, H). We do note that the low penetrant phenotypes caused by the *eor-1* alleles suggest that, while EOR-1 contributes to CCE, there must be other pathways that act in parallel.

**Fig. 3:**
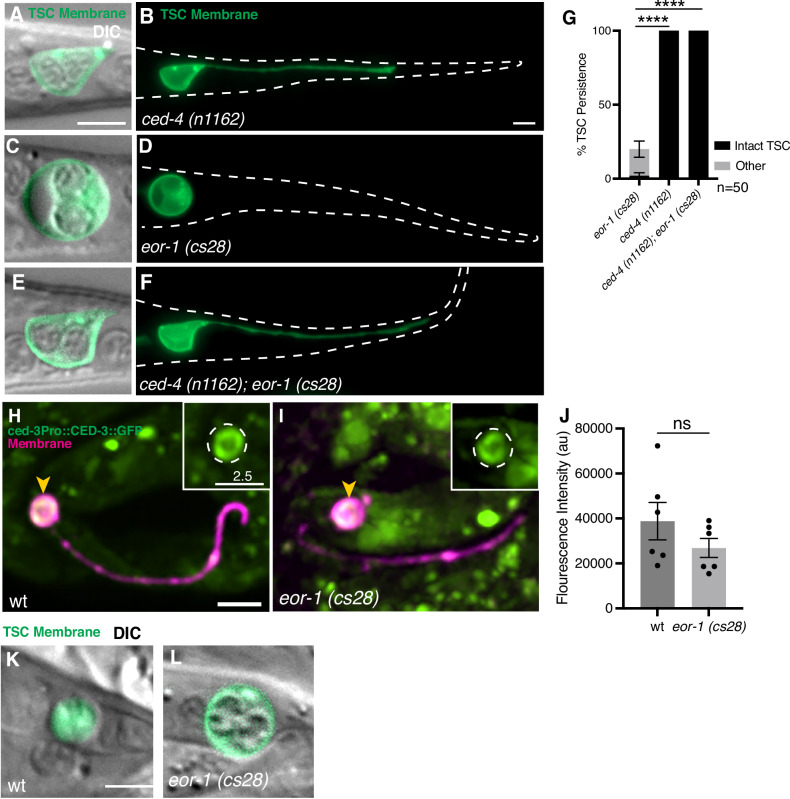
EOR-1/PLZF can function downstream of CED-3/Caspase. Intact TSC of *ced-4(n1162)* single mutants; **A** Merge of DIC and membrane GFP enlarged focusing on the soma; **B** Membrane GFP of whole cell view of (**A**). *n* = 10 biologically independent animals each with similar results. Soma-only persisting in *eor-1(cs28)* single mutant; **C** Merge of DIC and membrane GFP enlarged focusing on the soma; **D** Membrane GFP of whole cell view of (**C**). *n* = 10 biologically independent animals each with similar results. *ced-4(n1162); eor-1(cs28)* double mutant; **E** Merge of DIC and membrane GFP enlarged focusing on the soma; **F** Membrane GFP of whole cell view of (**E**). *n* = 10 biologically independent animals each with similar results. Green, TSC membrane. **G** Graph quantifying TSC persistence in (**A**–**F**). CED-3 promoter-driven translational fusion (green) against the TSC membrane (magenta), in wild type (**H**) *n* = 6, and *eor-1(cs28)* mutant (**I**) *n* = 6. **J** Fluorescence intensity graph for (**H**) and (**I**). Soma Distal Degrading (SDD) stage of TSC soma, merge of TSC membrane (green) and DIC in wild type (**K**) *n* = 10, and *eor-1(cs28)* mutant (**L**) *n* = 5. Scale bar, 5 μm, and 2.5 μm for inset of (**H–I**). Data are mean ± s.e.m. Statistics: two-tailed unpaired student’s *t* test, see Supplementary Table 3 for individual P-values.

Intrigued by the unusual soma/nuclear morphology in the *eor-1* mutant, we proceeded to examine TSC soma morphology across CCE steps incorporating DIC microscopy first in wild-type (Supplementary Fig. S2A–H”). While intact and prior to soma rounding, the TSC nuclei appear intact (Supplementary Fig. S2A–B”). At the beading stage (BA/BD), the soma rounds, and the nuclei begin to degrade (Supplementary Fig. S2C–D”). At the soma-distal process retracting stage (SDR), the soma forms a classic refractile, button-like appearance (Supplementary Fig. S2E–F”). After the SDR/retraction stage, and at the soma-distal degrading/SDD stage, the soma appears non-refractile with detached refractile bodies within the cytoplasm, and the membrane becomes diffuse, losing the discrete ring surrounding the cell (Fig. 3K and Supplementary Fig. S2G–H”).

We next examined CCE progression in the TSC soma under DIC in *eor-1(cs28)* mutants. We found that the TSC develops normally in *eor-1* mutants, as in wild-type (Supplementary Fig. S2I, J); and found that CCE progression appeared normal up until the SDD/soma-distal process degrading stage, where the nuclei fail to condense and the membrane does not lose its integrity (Fig. 3K, L and Supplementary Fig. S2O, P**”**). This suggests that EOR-1 may play a role in TSC soma removal at this late stage of CCE.

EOR-1 is the ortholog of the BTB/zinc-finger tumor-suppressor transcription factor PLZF implicated in the regulation of both apoptotic and non-apoptotic forms of PCD in *C. elegans* [34]. In the apoptotic death of the HSN neurons, EOR-1 has been reported to act upstream or in parallel to CED-9 [34]. In the non-apoptotic Linker-type Cell Death (LCD), EOR-1 has been reported to act in conjunction with the WNT signaling pathway [35]. The precise mechanistic contribution of EOR-1 to either form of PCD, however, remains unclear.

Given that prior studies for other forms of programmed cell death implicate EOR-1 in cell killing, and our observation of a TSC soma-specific defect in *eor-1* mutants, we were motivated to examine whether EOR-1 functions upstream of CED-3/caspase in an overall cell killing role, or downstream in either cell killing or clearance. In the absence of CED-3 and its upstream regulator CED-4, CCE fails to occur, leaving a fully intact TSC phenotype [30]. We generated a *ced-4(n1162); eor-1(cs28)* double mutant (Fig. 3E, F, G) and found an intact TSC and nuclear phenotype characteristic of *ced-4(n1162)* single mutants (*N* = 10/10) (Fig. 3A, B, G). This is distinct from *eor-1* single mutants (Fig. 3C, D, G) and suggests that EOR-1 may act downstream of CED-4 and CED-3 to promote CCE. We next examined whether *ced-3* transcription was affected in *eor-1* mutants by looking at a *ced-3* promoter-driven CED-3::GFP [42]. Prior work has shown that the PAL-1/Cdx-2 transcription factor is a direct transcriptional regulator for *ced-3* [33] in the TSC, such that loss of *pal-1* results in complete loss of the *ced-3* transcriptional signal, as visualized by a *ced-3* transcriptional reporter. While we do find CED-3::GFP signal in *eor-1* mutants, it is slightly decreased compared to wild type (Fig. 3H–J). As such, we cannot entirely rule out that EOR-1 does regulate CED-3 transcription in the TSC. However, as shown earlier, CCE is still able to proceed in *eor-1* mutants and the defect in CCE progression in *eor-1* mutants is during the SDD stage (Fig. 3K, L, Supplementary Fig. S1A–P**”**).

### EOR-1/PLZF promotes *wah-1*/Aif expression in the TSC

We next sought to identify the transcriptional target for EOR-1/PLZF in the context of CCE and examined published data obtained from a previous ChIP-Seq analysis of EOR-1/PLZF transcriptional targets [43]. We noted the gene *wah-1*, which encodes a homolog of the mitochondrial flavoprotein Apoptosis Inducing Factor AIF, a multifunctional enzyme that is essential for mitochondrial bioenergetic function [44]. Interestingly, WAH-1 has been shown to function to promote apoptotic cell death in the *C. elegans* germline [45, 46]. Upon activation by CED-3/Caspase and EGL-1/BH3-only, WAH-1/AIF promotes apoptosis through DNA degradation via CPS-6 endonuclease activation [46]. WAH-1/AIF has also been shown to aid in phosphatidylserine (PS) exposure by promoting SCRM-1 scramblase function [45] to facilitate apoptotic corpse recognition.

**Fig. 4:**
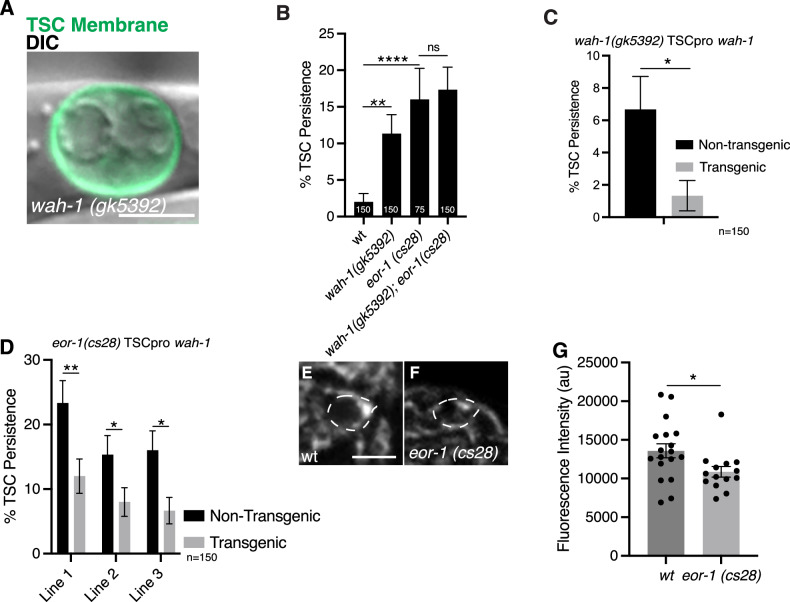
EOR-1/PLZF promotes *wah-1*/Aif expression in the TSC. **A** *wah-1(gk5392)* CCE defect of TSC soma persistence at L1. Merge of TSC membrane (green) and DIC. *n* = 10 biologically independent animals each with similar results. **B** Quantification of *wah-1* mutant defect compared to *eor-1(cs28)* and *wah-1(gk5392); eor-1(cs28)* double mutant. **C** Graph of TSC-specific rescue of *wah-1* mutant phenotype, *n* = 150. **D** Overexpression of *wah-1* in *eor-1(cs28)* biologically independent animals each with similar results. **E**–**F** Endogenous CRISPR GFP insertion for *wah-1* to evaluate *wah-1* expression in wild-type (**E**) and *eor-1(cs28)* (**F**) mutants. Images are sum projections at identical LUT values. **G** Quantification of GFP signal intensity across (**E**, **F**). Scale bar, 5 μm. Data are mean ± s.e.m. Statistics: two-tailed unpaired student’s *t* test, see Supplementary Table 3 for individual P-values.

We tested the hypothesis that EOR-1/PLZF promotes CCE through the transcriptional activation of *wah-1*. First, we assessed whether *wah-1* was required to promote CCE. We found that molecular null *wah-1*(*gk5392*) [47] mutants also contained persisting somas characteristic of *eor-1* mutants (Fig. 4A, B), and the double mutant is not additive (Fig. 4B). The double mutant combinations for *eor-1* and *wah-1* null mutants have similar proportions of defects as the single null mutants, suggesting these genes function in the same pathway. Cell-specific expression of *wah-1* in *wah-1(gk5392)* mutants rescues the *wah-1* mutant defect, suggesting that WAH-1, like EOR-1, acts in the TSC (Fig. 4C). We then overexpressed *wah-1* cDNA under the TSC promoter in *eor-1(cs28)* mutants and found that this construct rescued the CCE defect (Fig. 4D) o*f eor-1(cs28)* mutants, suggesting WAH-1 may act downstream of EOR-1. We next examined a CRISPR GFP insertion for *wah-1* at the intact mature (IMA) embryo stage in wild-type (Fig. 4E) and eor-1*(cs28)* (Fig. 4F) mutants and found decreased levels of *wah-1* expression in *eor-1(cs28)* mutants (Fig. 4G). As a control, we also examined *wah-1* expression in the germline and found no difference in intensity between wild-type and *eor-1(cs28)* mutants (Supplementary Fig. 3I–K). These data support the notion that WAH-1 promotes CCE by acting within the TSC and that it is transcriptionally regulated by EOR-1 in the TSC.

We next tested whether WAH-1 is a direct transcriptional target of EOR-1. We first addressed this in vivo by deleting the modEncode-reported 298 bp binding site of EOR-1 on the *wah-1* promoter [48] via CRISPR-Cas9. However, these animals were inviable, suggesting that this region is important for the role of WAH-1 in general development. We maintained the strain on a balancer, but this still yielded a very low number of mutant progeny that had no phenotype (*n* = 30). We next took an in vitro approach and attempted to express and purify EOR-1 protein for use in electrophoretic mobility shift assays (EMSA), but were unable to purify EOR-1. As such, we cannot definitively state that EOR-1 is a direct regulator of *wah-1* transcription, though our data does suggest at least an indirect level of positive regulation.

### EOR-1, WAH-1, and SCRM-1 promote corpse recognition and internalization

**Fig. 5:**
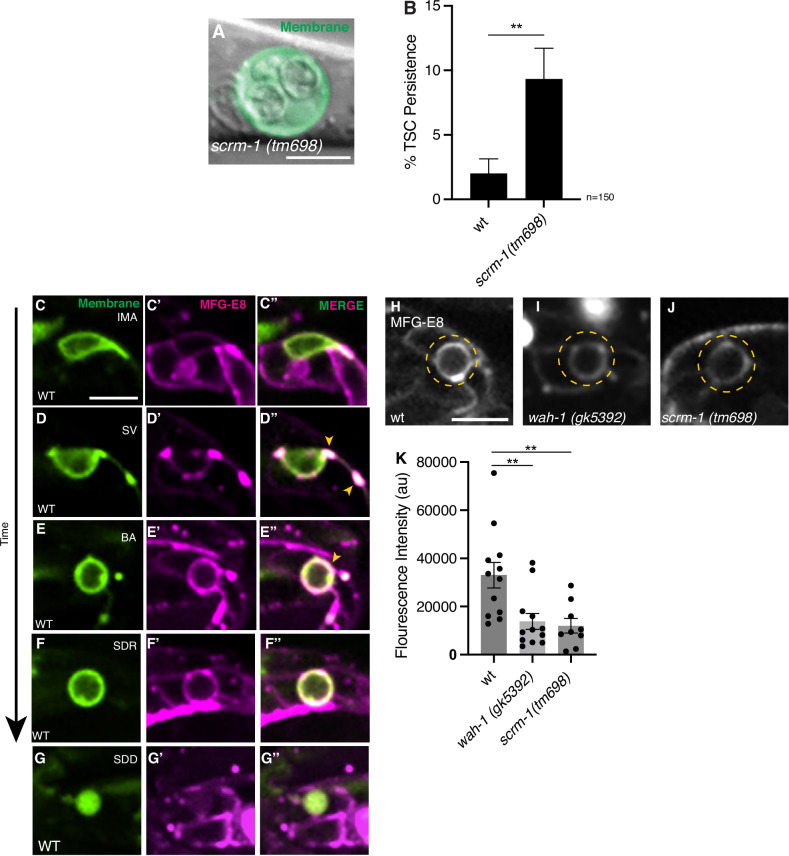
EOR-1 and WAH-1 promote corpse recognition. **A** *scrm-1(tm698)* mutant L1 showing persisting TSC soma and exaggerated nuclei. *n* = 9 biologically independent animals each with similar results. Merge of TSC membrane (green) and DIC. **B** Quantification of *scrm-1* mutant defect. **C–G”** *ced-1*pro::MFG-E8 sensor for phosphatidylserine (PS) in wild-type across CCE stages. PS reporter, magenta; TSC membrane, green. **C–C”** Intact mature (IMA) *n* = 10; **D–D”** Severing (SV) *n* = 6; **E–E”** Beading (BA) *n* = 12; **F–F”** Soma-distal retracting (SDR) *n* = 10; **G–G”** Soma Distal Degrading (SDD) *n* = 6. Arrows show PS presentation at soma-process junction and soma. **H–J** Comparison of PS presentation signal at the TSC beading (BA) stage in wild-type, *n* = 12; *wah-1(gk5932)*, *n* = 12; *and scrm-1(tm698)*, *n* = 9. Dotted line, outline of TSC soma. Images are sum projections at identical LUT values. **K** Quantification of (**H–J**). Scale bar, 5 μm. Data are mean ± s.e.m. Statistics: two-tailed unpaired student’s *t* test, see Supplementary Table 3 for individual P-values.

We next addressed how specifically EOR-1 and WAH-1 regulate CCE. Importantly, consistent with our observations for *eor-1* mutants, upon examination of CCE progression in *wah-1*(*gk5392*) mutants we found soma corpses arrest at the SDD stage (Supplementary Fig. S3G–H**”**). Prior studies have shown that WAH-1/AIF promotes apoptotic corpse recognition and engulfment by promoting SCRM-1 scramblase flipping of phosphatidylserine (PS) from the inner to the outer plasma membrane [45]. We found that *scrm-1(tm698)* mutants also displayed CCE defects (Fig. 5A, B) similar to *eor-1* and *wah-1* mutants. These data suggest that EOR-1 and WAH-1 may play roles in TSC soma corpse recognition via regulation of SCRM-1. This motivated us to look at the MFG-E8 PS reporter, which has been shown to label PS presented by both apoptotic and necrotic corpses [49] (Fig. 5C–G**”**). Interestingly, we did indeed see PS signal decorating the TSC soma at the severing stage (SV) (Fig. 5D–G**”**). We made an additional surprising, and intriguing observation-that PS exposure occurs with spatiotemporal specificity. Prior to PS exposure on the TSC soma, we observe signal at the soma-process junction, the membrane nicking-severing point at the start of CCE (Fig. 5D–D**”**). Future studies will explore the region-specific contribution of PS presentation to CCE.

Next, at the TSC soma, we compared PS exposure at the BA/BD stage in wild-type, *wah-1(gk5392)* and *scrm-1(tm698)* mutants and found the signal to be significantly lower in mutants (Fig. 5H–K). This suggests PS presentation at the TSC soma, like in the case of apoptosis, is dependent on WAH-1 and SCRM-1.

**Fig. 6:**
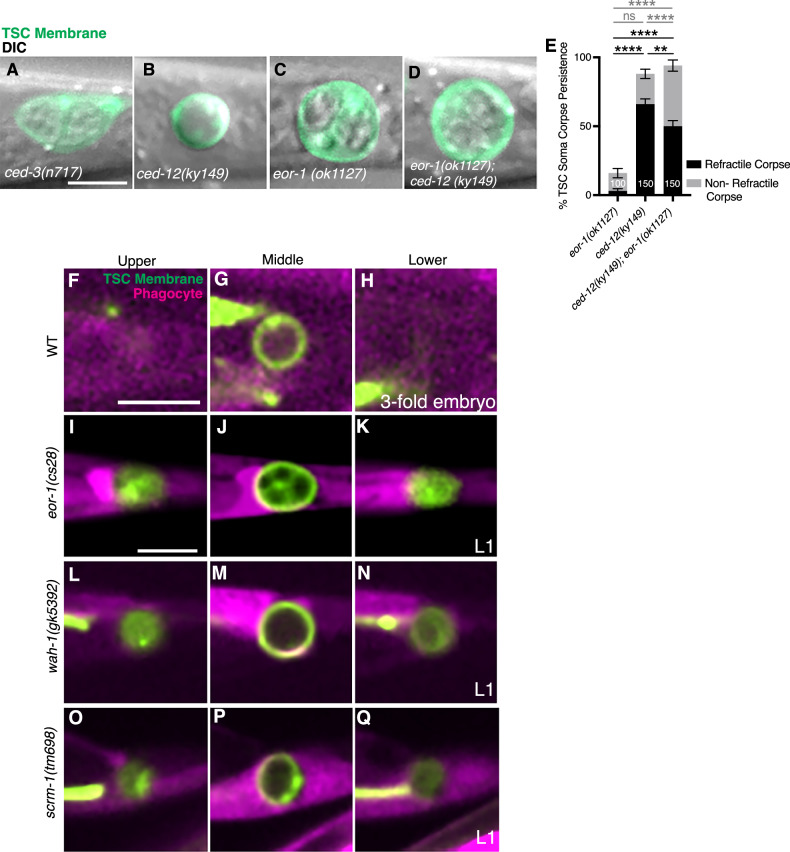
EOR-1, WAH-1, and SCRM-1 promote TSC corpse internalization. DIC and TSC membrane GFP merge view of TSC soma at L1 in *ced-3(n717)*, *n* = 10 (**A**); *ced-12(ky149)*, *n* = 5 (**B**); *eor-1(ok1127)*, *n* = 9 (**C**); *eor-1; ced-12*, *n* = 10 (**D**). **E** Quantification of TSC soma corpse only phenotype range as observed under DIC for (**B–D**), *ced-3(n717)* mutants (**A**) have a fully intact (soma and process) TSC phenotype. **F–Q** Assessment of TSC soma corpse (green, membrane) internalization into phagocyte (magenta) in L1 larvae in mutants at different planes of the phagocyte (upper, middle, lower): **F–H** Wild-type control, *n* = 4/4 internalized; **I–K** *eor-1(cs28)*, *n* = 10/15 not internalized; **L–N** *wah-1(gk5392)*, 12/17 not internalized; **O–Q** *scrm-1(tm698)*, 6/9 not internalized. Scale bar, 5 μm. Data are mean ± s.e.m. Statistics: two-tailed unpaired student’s *t* test, see Supplementary Table 3 for individual P-values.

We next compared the *eor-1(ok1127)* mutant defect with that of mutants for caspase/cell killing, *ced-3(n717)*, and engulfment, *ced-12(ky149)*, and performed epistasis experiments. We compared the soma-persistence defects of *ced-3(n717)* (Fig. 6A), *ced-12(ky149)* (Fig. 6B, E) and eor-1 mutants (Fig. 6C, E). We observed that the TSC somas of *eor-1(ok1127)* were morphologically distinct from both *ced-3(n717)* and *ced-12(ky149)* mutants. The *ced-12(ky149)* single mutant has a prominent phenotype of a refractile corpse, while the *eor-1(ok1127)* single mutant has a predominantly non-refractile phenotype. Unexpectedly, in a double mutant for *eor-1(ok1127)* and *ced-12(ky149)* (Fig. 6D, E), the proportion of non-refractile corpses increased compared to the single mutant of *ced-12(ky149)*. If the corpse was never internalized in absence of CED-12, we would expect the double to look like *ced-12(ky149)* with a comparable degree of refractile corpses. This confounding observation leads us to propose that the relationship between corpse engulfment and processing of the internalization may be more nuanced that currently appreciated. It will be interesting to explore this idea in future studies.

Comparing to wild-type in which the TSC soma corpse is internalized (Fig. 6F–H), we examined whether the persisting corpse of *eor-1(cs28)* (Fig. 6I–K and Supplementary Movie S1), *wah-1*(*gk5392*) (Fig. 6L–N and Supplementary Movie S2), and *scrm-1(tm698)* (Fig. 6O–Q, Supplementary Movie S3) mutants are internalized by its neighboring phagocyte. We used a cytosolic reporter labeling the TSC soma’s neighboring phagocyte (*skn-1p*::mKate2) and found that the persisting soma remains outside the phagocyte in these mutants, suggesting a failure of corpse internalization. The phagocyte pseudopods appeared not to be formed, suggesting failure in corpse recognition.

### WAH-1 also promotes corpse resolution post-internalization

WAH-1 has been shown to promote DNA degradation during apoptosis by acting to enhance the activity of the CPS-6/Endonuclease G [46, 50]. We tested whether this is true during CCE and attempted to separate WAH-1’s function in corpse internalization versus its possible nuclear role.

**Fig. 7:**
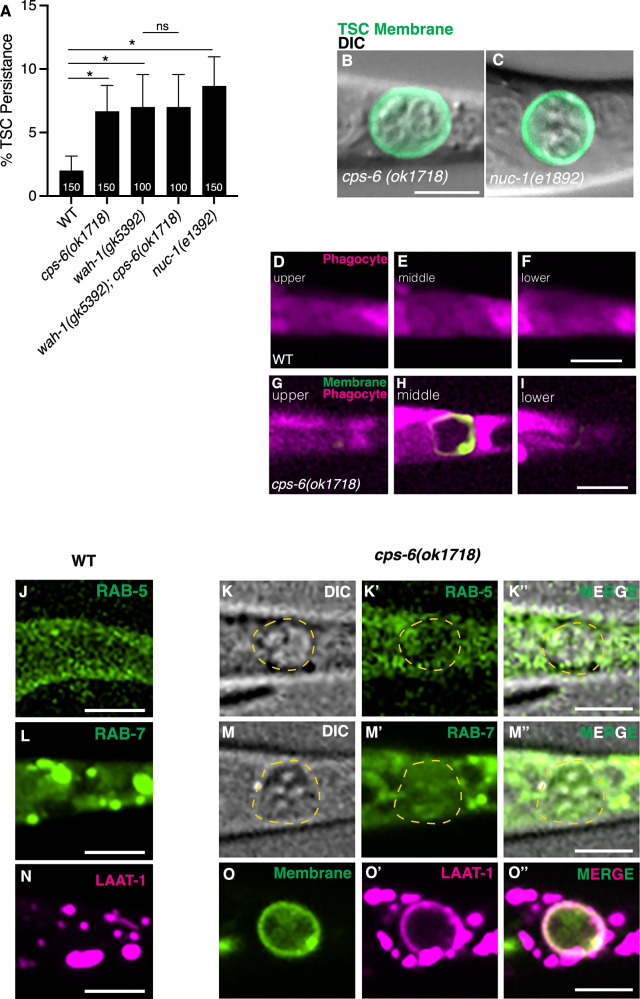
WAH-1 also promotes TSC soma corpse resolution post-internalization. **A** Quantification of *cps-6(ok1718)* and *nuc-1(e1382)* CCE defect of TSC (soma persistence) at L1. Images of DIC and TSC membrane GFP of *cps-6(ok1718)*, *n* = 6 (**B**); and *nuc-1(e1892)*, *n* = 10 (**C**). **D–F** Wild-type control for tests for soma corpse internalization across phagocyte planes (upper, middle lower), *n* = 4/4 internalized. **G–I** Test for soma corpse internalization for *cps-6(ok1718)* across phagocyte planes (upper, middle lower), *n* = 11/13 internalized. **J** Wild-type control for tests for localization of phagocyte GFP::RAB-5, *n* = 5. **K–K”** Localization of phagocyte GFP::RAB-5 relative to TSC corpse of *cps-6(ok1718)* mutant at L1 stage, *n* = 10. **L** Wild-type control for tests for localization of phagocyte GFP**::**RAB-7, *n* = 10. **M–M”** Localization of phagocyte GFP::RAB-7 relative to TSC corpse of *cps-6(ok1718)* mutant at L1 stage, *n* = 10. **N** Wild-type control for tests for localization of phagocyte LAAT-1::mCherry, *n* = 10. **O–O”** Localization of phagocyte LAAT-1::mCherry relative to TSC corpse of *cps-6(ok1718)* mutant at L1 stage, *n* = 10. Scale bar, 5 μm. Data are mean ± s.e.m. Statistics: two-tailed unpaired student’s *t* test, see Supplementary Table 3 for individual P-values.

We found that *cps-6(ok1718)* null mutants [37, 51] also have a persisting TSC soma similar to *wah-1* and *eor-1* mutants (Fig. 7A, B). Moreover, a *wah-1(gk5392); cps-6(ok1718)* double mutant did not show an increase in TSC defects (Fig. 7A), suggesting these genes function in the same genetic pathway. We also found that the null mutant, *nuc-1(e1392)* [52], for *nuc-1*, which encodes a lysosomal acid hydrolase that promotes late DNA degradation [52, 53], also has similar phenotypes to *eor-1* and *wah-1* mutants (Fig. 7A–C), supporting a role for WAH-1 in DNA degradation. We do note that the phenotypes of *cps-6* and *nuc-1* mutants are about 5%. As such, these genes can only have minor contributing functions, with other genes contributing to the process.

We investigated whether *cps-6* mutants arrested in the phagosome maturation stage. In wild-type worms the TSC soma corpse is internalized (Fig. 7D–F). Interestingly, unlike in *wah-1* and *eor-1* mutants, the soma of *cps-6* mutants appears to be internalized (Fig. 7G–I and Supplementary Movie S4) (*n* = 9/10), suggesting that CPS-6/EndoG is not involved in WAH-1’s corpse internalization function and, rather, likely plays a role in nuclear degradation downstream of internalization during phagosome maturation to resolve the internalized corpse.

To test in which stage of phagosome maturation WAH-1 and CPS-6 function together, we looked at known markers of phagosome maturation, comparing corresponding controls, for early (GFP::RAB-5) (Fig. 7J, K**”**), late (GFP::RAB-7) (Fig. 7L, M**”**) and phagolysosomes (LAAT-1::mCherry) (Fig. 7N, O**”**) [29] in *cps-6* mutants and in relevant wild type controls. We found that the phagosome bearing the internalized corpse is decorated by LAAT-1, suggesting arrest at the phagolysosomal stage; in contrast, RAB-5 and RAB-7 did not localize around the persisting soma. This is consistent with a role of CPS-6 in TSC DNA degradation. This also suggests that a defect in corpse degradation may contribute to the dramatic nuclear and cytoplasmic phenotypes in mutants.

**Fig. 8:**
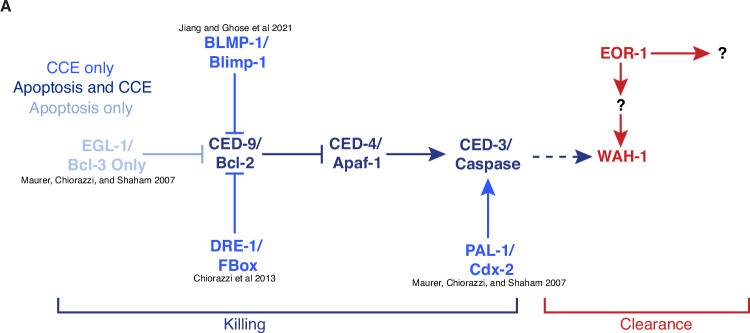
Model: EOR-1 promotes compartment-specific cell corpse clearance. **A** During Compartmentalized Cell Elimination (CCE), EOR-1 can act downstream of CED-3 caspase during TSC soma-specific clearance rather than an overall killing role as implied in other PCD studies. EOR-1 likely indirectly promotes *wah-1*/AIF expression to in turn promote both corpse recognition/internalization and phagosome maturation.

In summary, here we identify EOR-1 as a previously undescribed regulator of cell corpse clearance, acting in a compartment-specific manner during Compartmentalized Cell Elimination. During CCE, EOR-1 can function downstream of CED-3 caspase, and likely indirectly positively regulates *wah-1* (Fig. 8). This is, to our knowledge, the first report of EOR-1/PLZF in cell clearance. Our study offers mechanistic insights into the contribution of this master transcription factor in region-specific cell elimination during development, and also introduces new cell biological hallmarks of CCE.

## Discussion

Our study offers a mechanistic and cell-compartment-specific role of EOR-1 in promoting CCE that is distinct from other forms of PCD in *C. elegans*. While EOR-1 is implicated in regulating the onset of apoptotic death of the HSN neurons [34] and non-apoptotic LCD [35], we show that it plays a very different role in the TSC, promoting the phagocytic clearance of the cell’s own soma corpse.

Distinct from prior studies in other programmed cell death settings, we report here that EOR-1 can function downstream of CED-3. We speculate that EOR-1 may have a modified/repurposed role in PCD in the context of CCE owing to the independence of this cell death program from EGL-1.

Our work also suggests WAH-1 functions downstream of EOR-1 and that EOR-1 positively regulates WAH-1 transcription, but likely indirectly. We do note that deleting the modEncode-identified binding site for EOR-1 in the *wah-1* promoter [48] leads to inviable animals, suggesting EOR-1 may associate with WAH-1 in broader developmental roles. While we find EOR-1 does appear to regulate *wah-1* expression in the TSC, the disparate penetrance of TSC defects in *eor-1* and *wah-1* mutants suggest that there are other effectors or other transcriptional regulators that act downstream of EOR-1 to promote soma elimination during CCE.

Here we also report that PS is presented on the TSC at different compartments at different times. We show PS is presented first at the soma-process junction, where CCE is initiated as membrane nicking and severing, after which we observe PS on the TSC soma surface. Further studies will examine PS exposure in the different TSC compartments across space and time, and identify regulators of PS in these compartments. For example, is SCRM-1 also important for soma-process junction severing? Studies have shown that the ABC transporter CED-7 [54] is also involved in PS presentation during apoptotic cell clearance [49]. Does CED-7 have a compartment-specific role in PS presentation on the TSC? Our prior studies suggest that CED-7 has some involvement in both TSC soma and process clearance with mutants showing TSC persistence [29]. Whether it is related to region-specific PS presentation will be an exciting avenue to explore.

Our spatially and temporally higher resolution analysis allow us to describe the highly stereotyped morphological features of CCE in the soma compartment in detail. We suggest that failure to appropriately degrade dying cells can lead to severe exaggerations in nuclear morphology. We were surprised and intrigued to see similar defects in the TSC soma for *scrm-1* and *cps-6* mutants, previously shown to impact two different steps of the clearance of apoptotic cells—recognition versus digestion [45, 46]. It is not intuitive that a cell arrested in different parts of the cell elimination pathway, one outside the phagocyte and one inside, would have the same morphology. This will be an interesting direction for further study through both time-lapse imaging and higher resolution electron microscopy.

Taken together, our study expands our understanding of the transcriptional regulation of compartment-specific corpse clearance and provides mechanistic insights as to the roles for regulators of cell killing in broader cell elimination contexts, also highlighting new morphological hallmarks of programmed cell death.

## Materials and methods

### *C. elegans* methods

*C. elegans* strains were cultured using standard methods on *E. coli* OP50 and grown at 20 °C. Wild-type animals were the Bristol N2 subspecies. For most TSC experiments, one of two integrated reporters was used: *nsIs435* = TSCp::myrGFP or *nsIs685* = TSCp::mKate2-PH. Integration of extrachromosomal arrays was performed using UV and trioxsalen (T2137, Sigma). Animals were scored at 20 °C, unless otherwise mentioned.

### Imaging

Images were collected on a Nikon TI2-E Inverted microscope using a CFI60 Plan Apochromat Lambda 60× Oil Immersion Objective Lens, N.A. 1.4 (Nikon) and a Yokogawa W1 Dual Cam Spinning Disk Confocal. Images were acquired using NIS-Elements Advanced Research Package. For still embryo imaging, embryos were anesthetized using 0.5 M sodium azide. Larvae were paralyzed with 10 mM sodium azide. Widefield imaging was performed on a Carl Zeiss Axio Imager. M2 microscope with 63x oil-immersion lens.

### Quantifying CCE defects

TSC death defects were scored at the L1 stage. Animals were mounted on slides on 2% agarose-M9 pads, paralyzed with 10 mM sodium azide, and examined on a Carl Zeiss Axio Imager. M2 microscope with 63× oil immersion lens. The persisting TSC was identified by fluorescence based on its location and morphology. At least 50 animals were scored per genotype. For images, each image is representative of animals as indicated in the corresponding figure legend.

### Worm strains used in this study

List gene alleles under different chromosomes linkage groups (LG I-X)

LGI: *ced-12(ky149), mau-2(qm160), scrm-1(tm698), cps-6(ok1718)*

LGII:

LGIII*: ced-4(n1162), wah-1(gk5392)*

LGIV: *eor-1(ok1127), eor-1(cs28), ced-3(n717)*

LGV: *swsn-1(os22)*

LGX: *eor-2 (cs42), nuc-1(e1392)*

See Supplementary Table 2B for strain details.

### Plasmids and transgenics

Plasmids were generated via Gibson cloning. Primer sequences and information on the construction of plasmids used in this study are provided in Supplementary Table 1. The full list of transgenes is described in Supplementary Table 2. The full length or fragment of the *aff-1* promoter was used to label the TSC.

### CRISPR strain

A 298 bp region of the *wah-1* promoter was deleted (SUNY Biotech) from 5’-ttttcgaaatgtctatgaa-3’ to 3-tattctacctgaaatg-5’ to generate PHX10705 (confirmed via Wormbase) [55].

### Quantification of WAH-1::GFP fluorescence intensity

Images of the intact TSC in animals harboring *wah-1* promoter driven *wah-1*::GFP in WT and *eor-1(cs28)*, were collected using confocal microscopy as stated above using the same settings across all images. Sum intensity projections of the TSC soma were generated by following TSCp::mCherry in Fiji Image J software, and GFP intensity was then calculated using this software. CTCF was calculated using Microsoft Excel and graphed using GraphPad. All data points represent the average of 10 animals. Statistical significance was determined by a two-tailed unpaired Student’s *t* test for comparison between wild-type and mutant animals.

### Statistics

Sample sizes and statistics were based on previous studies of CCE and the TSC with similar methodologies) [29, 32, 56–58]. All experiments were repeated at least two to three times, as indicated, giving similar results. Independent transgenic lines were treated as independent experiments. An unpaired two-tailed t-test was used for all persisting TSC quantifications (GraphPad Prism). For all figures, mean ± standard error of the mean (s.e.m.) is represented. For both Main and Supplementary Figures, sample sizes are indicated directly in the figure for graphs and in the legend for images. P-values are indicated in Supplementary Table 3.

## Supplementary information

Supplementary Figure S1

Supplementary Figure S2

Supplementary Figure S3

Supplementary Table 1

Supplementary Table 2

Supplementary Table 3

Movie M1

Movie M2

Movie M3

Movie M4

Supplementary Material Legends
