# Supplementary figures and images for: EOR-1/PLZF promotes WAH-1/AIF-dependent compartment-specific corpse clearance
Source: Cell Death Discov. 2025 Nov 28;12:23. doi: 10.1038/s41420-025-02874-2 (PMC12808754; doi:10.1038/s41420-025-02874-2)

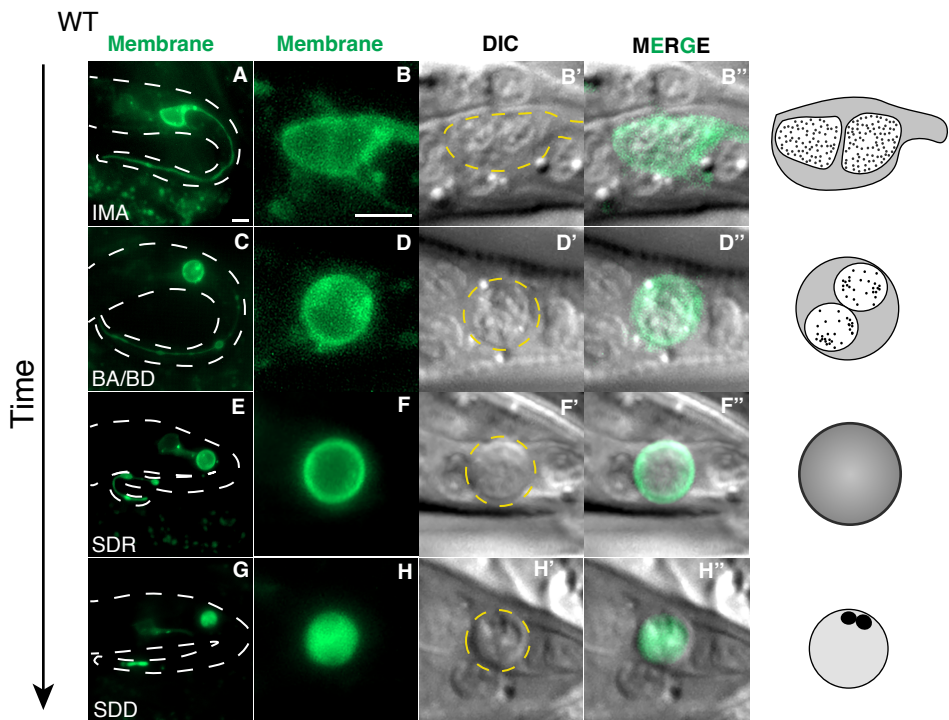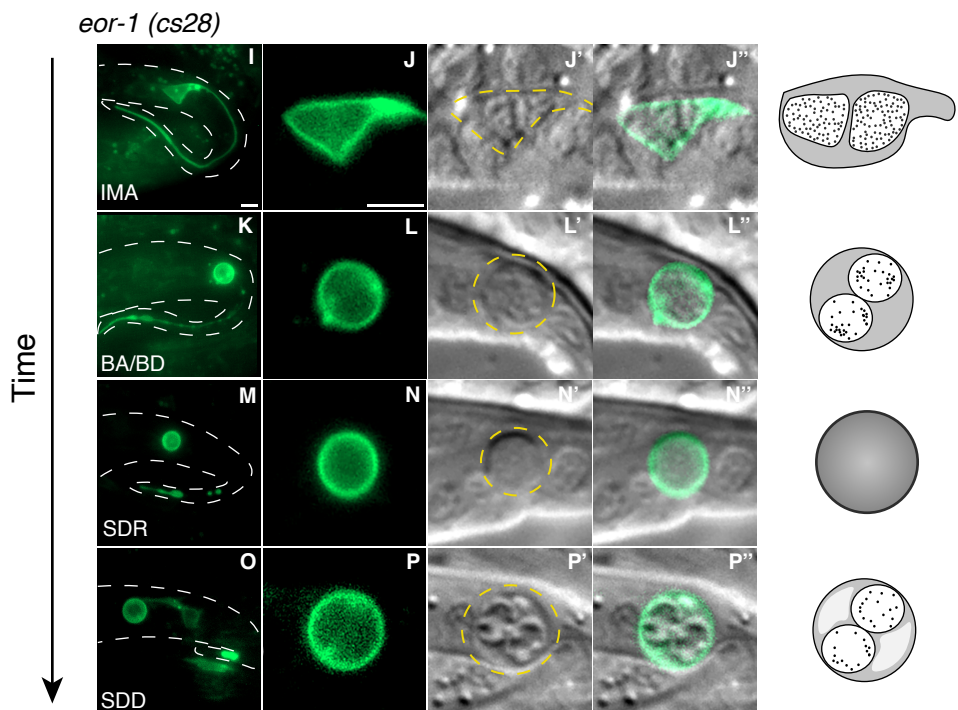

Supplement: Supplementary file 1 — Supplementary Figure S1 [file 41420_2025_2874_MOESM1_ESM.pdf]

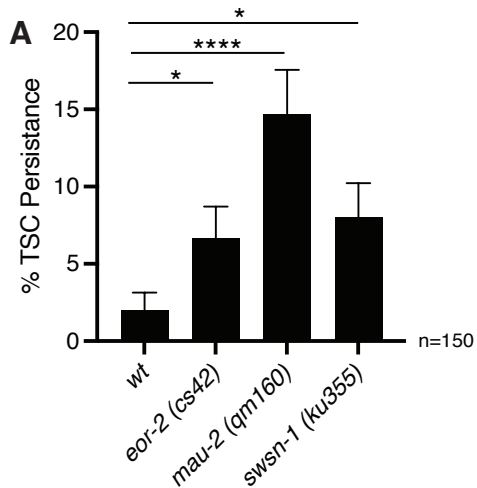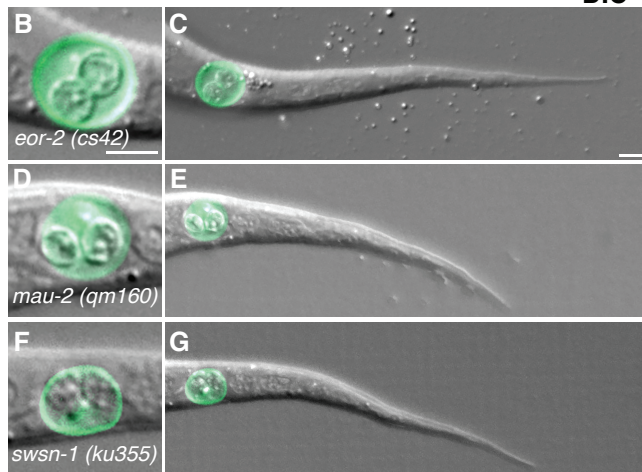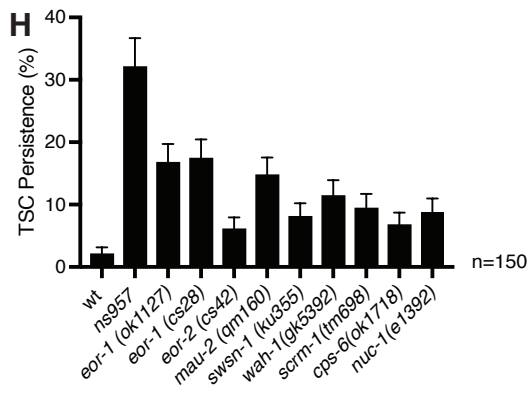

Supplement: Supplementary file 2 — Supplementary Figure S2 [file 41420_2025_2874_MOESM2_ESM.pdf]

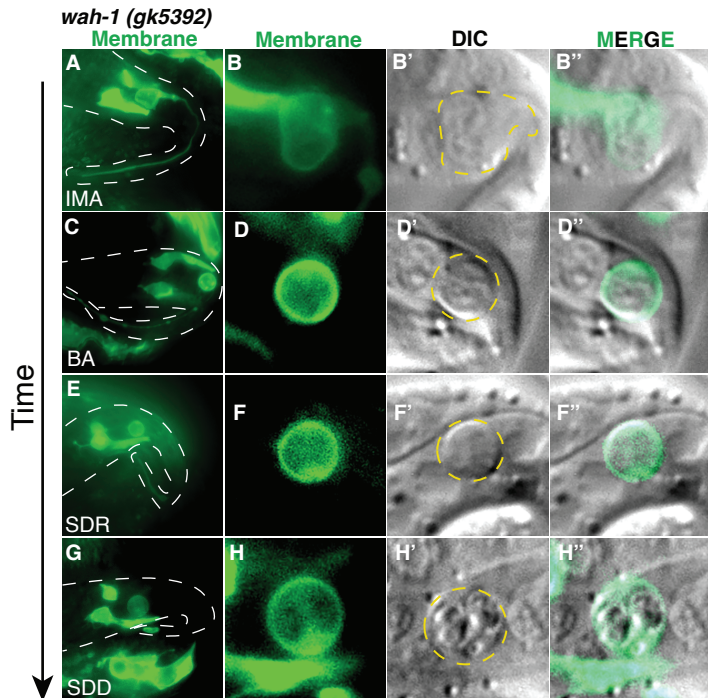

### WAH-1::GFP

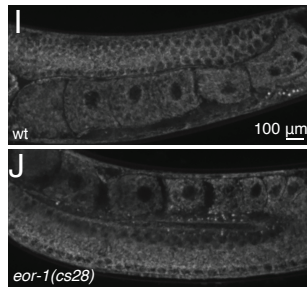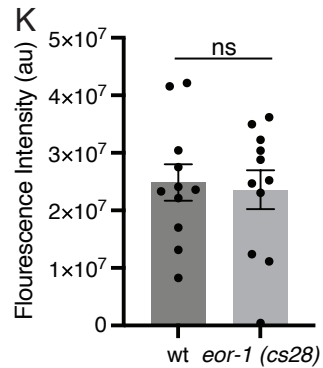

Supplement: Supplementary file 3 — Supplementary Figure S3 [file 41420_2025_2874_MOESM3_ESM.pdf]
