# Supplementary material for: EOR-1/PLZF promotes WAH-1/AIF-dependent compartment-specific corpse clearance
Source: Cell Death Discov. 2025 Nov 28;12:23. doi: 10.1038/s41420-025-02874-2 (PMC12808754; doi:10.1038/s41420-025-02874-2)
Supplement: Supplementary file 5 — Supplementary Table 2 [file 41420_2025_2874_MOESM5_ESM.docx]

| **Strain name (TSC #)** | **Genotype** | **Comments** |
| --- | --- | --- |
| OS8095 | *ced-3 (n717); nsls435* | nsIs435 = aff1p::myrGFP |
| TSC293 | *eor-1(ok1127); nsIs435* | nsIs435 = aff1p::myrGFP |
| TSC307 | *ns957; nsIs435 eor-1 fosmid (WRM0616aA08) rescue Line 1* | nsIs435 = aff1p::myrGFP |
| TSC308 | *ns957; nsIs435 eor-1 fosmid (WRM0616aA08) rescue Line 2* | nsIs435 = aff1p::myrGFP |
| TSC313 | *ns957; nsIs435 eor-1 fosmid (WRM0616aA08) rescue Line 3* | nsIs435 = aff1p::myrGFP |
| TSC363 | *unc-119(ed3) III; wgIs350(eor-1::GFP);nsIs686* | nsIs686 = pPG196 pPG196 = pSM_aff1p(small)_mKate2PHutr |
| TSC364 | *ced-12(k149);nsIs435* | nsIs435 = aff1p::myrGFP |
| TSC367 | *ced-12(k149); eor-1 (ok1127); nsIs435* | nsIs435 = aff1p::myrGFP |
| TSC376 | *mau-2(qm160); nsIs435* | nsIs435 = aff1p::myrGFP |
| TSC377 | *swsn-1(ku355); nsIs431* | nsIs435 = aff1p::myrGFP |
| TSC381 | *eor-2(cs42); nsIs435* | nsIs435 = aff1p::myrGFP |
| TSC426 | *eor-1 (cs28); nsIs435* | nsIs435 = aff1p::myrGFP |
| TSC434 | *eor-1 (cs28); nsIs528* | nsIs528 = [aff1p(4 to 2.75)::myrGFP];WT; from pPG99 |
| TSC490 | *wah-1 (gk5392); nsIs435* | nsIs435 = aff1p::myrGFP |
| TSC597 | *eor-1(cs28); wah-1(gk5392); nsIs435* | nsIs435 = aff1p::myrGFP |
| TSC602 | *eor-1(cs28); ced-4 (n1162); nsls435* | nsIs435 = aff1p::myrGFP |
| TSC614 | *cps-6 (ok1718); nsls435* | nsIs435 = aff1p::myrGFP |
| TSC615 | *nuc-1 (e1392); nsls435* | nsIs435 = aff1p::myrGFP |
| TSC658 | *eor-1(cs28); ppg425; nsls435; mccEx265* | mccEx265= pPG146 =aff1p::eor-1 |
| TSC659 | *eor-1(cs28); ppg425; nsls435; mccEx266* | mccEx265= pPG146 =aff1p::eor-1 |
| TSC660 | *eor-1(cs28); ppg425 5.0ng; nsls435; mccEx267* | mccEx265= pPG146 =aff1p::eor-1 |
| TSC670 | *eor-1 (cs28); nsIs435;mccEx096* | mccEx096=pPG312: 20ng/uL, myo-2GFP: 5ng/uL, pBSK: 80ng/uL; nsIs435=aff-1p(small)myrGFP. skn-1 expression (pPG312 injected into OS8095. pPG312: 20ng/uL, myo-2GFP: 5ng/uL, pBSK: 80ng/uL) |
| TSC676 | *scrm-1(tm698); nsls435* | nsIs435 = aff1p::myrGFP |
| TSC678 | *cps-6 (ok1718); wah-1 (gk5392); nsls435* | nsIs435 = aff1p::myrGFP |
| TSC680 | *wah-1(gk5392; pPG460 1.0ng line 2; nsls435; mccEx275* | pPG460=aff1p::wah-1 mccEx275 nsIs435 = aff1p::myrGFP |
| TSC694 | *cps-6 (ok1718); nsIs435;mccEx096* | mccEx096=pPG312=skn-1 expression mKate2 nsIs435=aff-1p(small)myrGFP. |
| TSC695 | *cps-6 (ok1718); nsIs435;nsEx5971* | nsEx5971 = ced-1p::LAAT-1::mCherry + coel-GFP nsIs435 = aff1p::myrGFP +coel-red |
| TSC696 | *cps-6 (ok1718);rab-7(utx12[mNG::rab-7])* | Superficially wild-type. N-terminal tag of RAB-7 via CRISPR/Cas9 knock-in of mNeonGreen at rab-7 locus. Insertion verified by PCR. Left flank: 5' gcacaacaaaaaggcttccagtgaacaaaa 3'; Right flank: 5' ATGTCGGGAACCAGAAAGAAGGCGCTGCTC 3'. sgRNA: 5' cttccagtgaacaaaaATGT 3'. CRISPR/Cas9 homologous recombination, not outcrossed. |
| TSC698 | *wah-1(gk5392); nsIs435;mccEx280* | mccEx280=pPG312=skn-1 expression mKate2 nsIs435=aff-1p(small)myrGFP. |
| TSC703 | *nsIs435;mccEx280* | mccEx096=pPG312=skn-1 expression mKate2 nsIs435=aff-1p(small)myrGFP. |
|  |  |  |
| TSC698 | *wah-1(gk5392); nsIs435;mccEx280* | mccEx280=pPG312: 20ng/uL, no co-injection marker; nsIs435=aff-1p(small)myrGFP. |
| TSC703 | *nsIs435;mccEx280* | mccEx280=pPG312; nsIs435=aff-1p(small)myrGFP. |
| TSC705 | *cps-6 (ok1718);rab-5(udn14); udnSi38* | udnSi38 [rab5p::rab-5] II. rab-5 [D135H]. rab-5 variant edit #2. Homozygous lethal rab-5 [D135H] mutation rescued by a single copy of wild-type rab-5 integrated into chromosome II at ttTi5605 site (II: 0.77). Maintain at 20 degrees. Reference: Huang et al. 2022. PMID: 35121658. CRISPR/Cas9 mutagen, not outcrossed. |
| TSC718 | *wah-1(mcc36)[WAH-1::GFP]; nsIs686* | mcc36=wah-1(mcc36)[WAH-1::GFP]; nsIs686 = pPG196+coel-red line 30.1 pPG196 = pSM_aff1p(small)_mKate2PHutr; probably on X. CRISPR mutant, GFP insertion after stop. |
| TSC839 | *smIs10 I; (eor-1 cs28)* | smIs10 [ced-3p::ced-3::GFP + rol-6(su1006)] I. Rollers. Reference: Geng X, et al. Nat Struct Mol Biol. 2008 Oct;15(10):1094-101. Gamma ray mutagen, outcrossed 8x's. |
| TSC840 | *wah-1(mcc36)[WAH-1::GFP]; nsIs686; eor-1 (cs28)* | mcc36=wah-1(mcc36)[WAH-1::GFP]; nsIs686 = pPG196 = pSM_aff1p(small)_mKate2PHutr; probably on X. CRISPR mutant, GFP insertion after stop. |
| TSC841 | *eor-1 (cs28); pPG460 10ng line 1; nsls435;* | mccEx274= co injected green myo nsIs435 = aff1p::myrGFP + coel-red probably on V, pPG460=TSCp wah-1 rescue |
| TSC842 | *eor-1 (cs28); pPG460 5ng line 2; nsls435;* | mccEx274= co injected green myo nsIs435 = aff1p::myrGFP + coel-red probably on V, pPG460=TSCp wah-1 rescue |
| TSC843 | *eor-1 (cs28); pPG460 5ng line 3; nsls435;* | mccEx274= co injected green myo nsIs435 = aff1p::myrGFP + coel-red probably on V, pPG460=TSCp wah-1 rescue |

**Supplementary Table 2B.**

**Summary of alleles:**

| **Allele** | **Description** | **Reference** |
| --- | --- | --- |
| *ced-12(ky149)* | Point mutation R38>STOP, likely null. | (1) |
| *mau-2(qm160)* | Molecular null. | (2) |
| *scrm-1(tm698)* | Deletion in last intron + exon. | (3) |
| *cps-6(ok1718)* | Estimated deletion of 676 bp from 5’-CCTTCCCCTATTTCGGATGAATTTTTGTTG-3’ to 5’-AGTTACGTGTTTTTGCGAAAAACTTCGTCG-3’.  Presumed null; 100% of dopaminergic neurons survive due to a-Synuclein production versus only 50% in wild-type. | (4, 5) |
| *ced-4(n1162)* | Stop codon at amino acid 80*.* | (6) |
| *wah-1(gk5392)* | 9220 bp deletion from 5’-TGTTTACACGCCCACCAATCTTCCCCGCCC-3’ to 5’-TTCGGACCTTCACTGGATGTAGCTCGGCGT-5’.  Presumed null mutant owing to large deletion and sterility as homozygote. | (7) |
| *eor-1(ns957)* | This study; E570K change in the Zn finger domain. |  |
| *eor-1(ok1127)* | Estimated 1200 bp deletion from 5’-GAAGTTGCTGGAGTTGAGCC-3’ to 5’-CTTTGCCGAAGGAAACACAT-3’.  Null mutant | (4, 8) |
| *eor-1(cs28)* | 68-bp dele*tion* from exons 5 and 6 that leads to a frameshift.  Null mutant | (9, 10) |
| *ced-3(n717)* | The true ced-3 null allele not officially defined. Considered loss-of-function mutants have been described in detail. Contains mutation of the conserved acceptor site of intron 7. | (11) |
| *swsn-1(os22)* | Mutation of GTC/CCC/TCA to GTC/CTC/TCA causing a P86L substitution. | (12) |
| *eor-2 (cs42)* | Predicted to encode a truncated protein of only 40 amino acids, Q41stop at exon 2. | (9) |
| *nuc-1(e1392)* | G-to-A transition predicted to convert Trp59 to a TAG (amber) stop codon. | (13) |
| PHX10705 | A 298 bp region of the *wah-1* promoter was deleted (SUNY Biotech) from 5’-ttttcgaaatgtctatgaa-3’ to 3-tattctacctgaaatg-5’ to generate PHX10705 confirmed via Wormbase (14). | (14) |

**References**

1. Wu YC, Tsai MC, Cheng LC, Chou CJ, Weng NY. C. elegans CED-12 acts in the conserved crkII/DOCK180/Rac pathway to control cell migration and cell corpse engulfment. Dev Cell. 2001;1(4):491-502.

2. Benard CY, Kebir H, Takagi S, Hekimi S. mau-2 acts cell-autonomously to guide axonal migrations in Caenorhabditis elegans. Development. 2004;131(23):5947-58.

3. Wang X, Wang J, Gengyo-Ando K, Gu L, Sun CL, Yang C, et al. C. elegans mitochondrial factor WAH-1 promotes phosphatidylserine externalization in apoptotic cells through phospholipid scramblase SCRM-1. Nat Cell Biol. 2007;9(5):541-9.

4. Consortium CeDM. large-scale screening for targeted knockouts in the Caenorhabditis elegans genome. G3 (Bethesda). 2012;2(11):1415-25.

5. Buttner S, Habernig L, Broeskamp F, Ruli D, Vogtle FN, Vlachos M, et al. Endonuclease G mediates alpha-synuclein cytotoxicity during Parkinson's disease. EMBO J. 2013;32(23):3041-54.

6. Chen L, McCloskey T, Joshi PM, Rothman JH. ced-4 and proto-oncogene tfg-1 antagonistically regulate cell size and apoptosis in C. elegans. Curr Biol. 2008;18(14):1025-33.

7. Au V, Li-Leger E, Raymant G, Flibotte S, Chen G, Martin K, et al. CRISPR/Cas9 Methodology for the Generation of Knockout Deletions in Caenorhabditis elegans. G3 (Bethesda). 2019;9(1):135-44.

8. Yemini E, Lin A, Nejatbakhsh A, Varol E, Sun R, Mena GE, et al. NeuroPAL: A Multicolor Atlas for Whole-Brain Neuronal Identification in C. elegans. Cell. 2021;184(1):272-88 e11.

9. Howard RM, Sundaram MV. C. elegans EOR-1/PLZF and EOR-2 positively regulate Ras and Wnt signaling and function redundantly with LIN-25 and the SUR-2 Mediator component. Genes Dev. 2002;16(14):1815-27.

10. Shinkai Y, Kuramochi M, Doi M. Regulation of chromatin states and gene expression during HSN neuronal maturation is mediated by EOR-1/PLZF, MAU-2/cohesin loader, and SWI/SNF complex. Sci Rep. 2018;8(1):7942.

11. Shaham S, Reddien PW, Davies B, Horvitz HR. Mutational analysis of the Caenorhabditis elegans cell-death gene ced-3. Genetics. 1999;153(4):1655-71.

12. Smith JJ, Xiao Y, Parsan N, Medwig-Kinney TN, Martinez MAQ, Moore FEQ, et al. The SWI/SNF chromatin remodeling assemblies BAF and PBAF differentially regulate cell cycle exit and cellular invasion in vivo. PLoS Genet. 2022;18(1):e1009981.

13. Wu YC, Stanfield GM, Horvitz HR. NUC-1, a caenorhabditis elegans DNase II homolog, functions in an intermediate step of DNA degradation during apoptosis. Genes Dev. 2000;14(5):536-48.

14. Sternberg PW, Van Auken K, Wang Q, Wright A, Yook K, Zarowiecki M, et al. WormBase 2024: status and transitioning to Alliance infrastructure. Genetics. 2024;227(1).
