# Supplementary material for: EOR-1/PLZF promotes WAH-1/AIF-dependent compartment-specific corpse clearance
Source: Cell Death Discov. 2025 Nov 28;12:23. doi: 10.1038/s41420-025-02874-2 (PMC12808754; doi:10.1038/s41420-025-02874-2)
Supplement: Supplementary file 6 — Supplementary Table 3 [file 41420_2025_2874_MOESM6_ESM.docx]

| **Figure Panel** | **t-test comparison** | **P Value** |
| --- | --- | --- |
| 2 D | wt 20C vs *ns957* 20C | <0.0001 |
| 2 D | wt 25C vs ns957 25C | <0.0001 |
| 2 E | wt 20C vs eor-1(ns957) 20C | 0.0002 |
| 2 E | wt 20C vs eor-1(ok1127) 20C | 0.0012 |
| 2 E | wt 20C vs eor-1(cs28) 20C | 0.0040 |
| 2 E | eor-1(ns957) 20C vs eor-1(ok1127) 20C | 0.5167 |
| 2 E | eor-1(ns957) 20C vs eor-1(cs28) 20C | 0.2594 |
| 2 E | eor-1(ok1127) 20C vs eor-1(cs28) 20C | 0.6282 |
| 2 E | wt 25C vs eor-1(ns957) 25C | <0.0001 |
| 2 E | wt 25C vs eor-1(ok1127) 25C | <0.0001 |
| 2 E | wt 25C vs eor-1(cs28) 25C | <0.0001 |
| 2 E | eor-1(ns957) 25C vs eor-1(ok1127) 25C | 0.0005 |
| 2 E | eor-1(ns957) 25C vs eor-1(cs28) 25C | 0.0009 |
| 2 E | eor-1(ok1127) 25C vs eor-1(cs28) 25C | 0.8697 |
| 2 F | Line 1 Transgenic vs Line 1 Control | <0.0001 |
| 2 F | Line 2 Transgenic vs Line 2 Control | <0.0001 |
| 2 F | Line 3 Transgenic vs Line 3 Control | <0.0001 |
| 2 H | Line 1 Transgenic vs Line 1 Control | 0.0012 |
| 2 H | Line 2 Transgenic vs Line 2 Control | 0.0294 |
| 2 H | Line 3 Transgenic vs Line 3 Control | 0.0008 |
| 3 G | eor-1(cs28) vs ced-4(n1162) | <0.0001 |
| 3 G | eor-1(cs28) vs ced-4(n1162); eor-1(cs28) | <0.0001 |
| 3 J | wt vs *eor-1 (cs28)* | 0.2291 |
| 4 B | wt vs *wah-1 (gk5392)* | 0.0011 |
| 4 B | wt vs *eor-1 (cs28)* | <0.0001 |
| 4 B | *wah-1(gk5392)*; *eor-1(cs28)* vs *eor-1 (cs28)* | 0.983 |
| 4 C | Transgenic vs Control | 0.0184 |
| 4 D | Line 1 Transgenic vs Line 1 Control | 0.01 |
| 4 D | Line 2 Transgenic vs Line 2 Control | 0.0481 |
| 4 D | Line 3 Transgenic vs Line 3 Control | 0.0107 |
| 4 H | wt vs *eor-1 (cs28)* | 0.0306 |
| 5 B | wt vs scrm-1(tm698) | 0.0059 |
| 5 K | wt vs wah-1 (gk5392) | 0.0058 |
| 5 K | wt vs scrm-1 (tm698) | 0.0055 |
| 6 E | eor-1(ok1127) vs ced-12(ky149) Refractile | <0.0001 |
| 6 E | eor-1(ok1127) vs eor-1(ok1127);ced-12(ky149) Refractile | <0.0001 |
| 6 E | ced-12(ky149) vs eor-1(ok1127);ced-12(ky149) Refractile | 0.0049 |
| 6 E | eor-1(ok1127) vs ced-12(ky149) Non-Refractile | 0.0725 |
| 6 E | eor-1(ok1127) vs eor-1(ok1127);ced-12(ky149) Non-Refractile | <0.0001 |
| 6 E | ced-12(ky149) vs eor-1(ok1127);ced-12(ky149) Non-Refractile | <0.0001 |
| 7A | wt vs *cps-6 (ok1718)* | 0.0473 |
| 7A | wt vs *wah-1 (gk5392)* | 0.0484 |
| 7A | *wah-1(gk5392)* vs *wah-1(gk5392)*; *cps-6 (ok1718)* | >0.9999 |
| 7A | wt vs *nuc-1 (e1392)* | 0.0101 |
| S 2 A | wt vs *eor-2 (cs42)* | 0.0473 |
| S 2 A | wt vs *mau-2 (qm160)* | <0.0001 |
| S 2 A | wt vs *swsn-1 (ku355)* | 0.0061 |
| S 3 L | wt vs *eor-1 (cs28)* | 0.7909 |
