# Supplementary material for: EOR-1/PLZF promotes WAH-1/AIF-dependent compartment-specific corpse clearance
Source: Cell Death Discov. 2025 Nov 28;12:23. doi: 10.1038/s41420-025-02874-2 (PMC12808754; doi:10.1038/s41420-025-02874-2)
Supplement: Supplementary file 11 — Supplementary Material Legends [file 41420_2025_2874_MOESM11_ESM.docx]

**Supplemental Data**

**Supplemental Tables:**

**Supplemental Table 1.** Plasmids used in this study.

**Supplemental Table 2.** Strains and Transgenes used in this study.

**Supplemental Table 3.** Statistics and P-values.

**Supplemental Figures**

**Supplemental Figure S1. EOR-1/PLZF functions in CCE with its known partners. (A)** CCE defects across *eor-1* alleles and *eor-2*, *mau-2* and *swsn-1* mutants. **(B, C)** persisting TSC soma phenotype of *eor-2(cs42)* single mutants. (**B**) Merge of DIC and membrane GFP enlarged focusing on the soma, (**C**) whole cell view of (**B**). n=10 biologically independent animals each with similar results. **(D, E)** Soma-only persisting in *mau-2(qm160)* single mutant. (**D**) Merge of DIC and membrane GFP enlarged focusing on the soma, (**E**) whole cell view of (**C**). n=10 biologically independent animals each with similar results. **(F, G)** persisting TSC soma phenotype of *swsn-1(ku355)* mutants. (**F**) Merge of DIC and membrane GFP enlarged focusing on the soma, (**G**) whole cell view of (**F**). n=10 biologically independent animals each with similar results. Scale bar, 5μm. (**H**) Combined graph representing (%) TSC persistence from figures 2D for wt, 2C for *ns957*, 2D for *eor-1 (ok1127)* and *eor-1 (cs28)*, S2 A for *eor-2 (cs42)*, *mau-2 (qm160)*, and *swsn-1 (ku355)*, 4B for *wah-1 (gk5392)*, 5B for *scrm-1 (tm698)*, and 7A for *cps-6 (ok1718)* and *nuc-1 (e1392).* For statistics see corresponding main figure.

**Supplemental Figure S2. TSC soma morphological changes across CCE stages in wild-type and *eor-1* mutants with corresponding schematics.** CCE of TSC (membrane marker, green) in wild-type **(A-H'')** and *eor-1(cs28)* **(I-P’’)** mutants showing progression of soma elimination. Left-most panels show whole cell. Wild type (**A, C, E, G**) are the same images as presented in **Fig 1** **(A-E).** (**A-B''**) IMA stage in wild-type, n=10 biologically independent animals each with similar results, (**C-D''**) BA/BD stage in wild-type, n=8 biologically independent animals each with similar results, (**E-F''**) SDR stage in wild-type, n=10 biologically independent animals each with similar results, (**G-H''**) SDD stage in wild-type, n=10 biologically independent animals each with similar results. (**I-J''**) *eor-1(cs28)* IMA, n=8 biologically independent animals each with similar results, (**K-L''**) *eor-1(cs28)* BA/BD, n=6 biologically independent animals each with similar results, (**M-N''**) *eor-1(cs28)* SDR, n=10 biologically independent animals each with similar results, (**O-P''**) *eor-1(cs28)* SDD, n = 5 abnormal/SDD normal biologically independent animals each with similar results. Scale bar, 5μm. Yellow dotted line, soma outline.

**Supplemental Figure S3. CCE progression in *wah-1(gk5392)* and quantification of WAH-1::GFP in the germline. (A-B'')** TSC membrane, green. IMA *wah-1(gk5392),* n=4 biologically independent animals each with similar results, **(C-D'')** BA *wah-1(gk5392),* n=5 biologically independent animals each with similar results, **(E-F'')** SDR *wah-1(gk5392),* n=4 biologically independent animals each with similar results, **(G-H'')** SDD *wah-1(gk5392),* n=3 biologically independent animals each with similar results. **(I-K)** Control images of germline WAH-1::GFP CRISPR insertion in wild-type, n= 11 (**I**); *eor-1(cs28),* n=11 (**J**); (**K**). **(L)** Quantification of germline rachis. Data are mean ± s.e.m. Statistics: two-tailed unpaired student's *t*-test, see **Supplementary Table 3** for individual P-values. Scale bar, 5μm.

**Supplemental Movies:**

**Supplemental Movie S1. Test for TSC soma internalization of *eor-1(cs28)* mutants.** Green, TSC membrane. Magenta, phagocyte cytosolic marker.

**Supplemental Movie S2. Test for TSC soma internalization of *wah-1(gk5392)* mutants.** Green, TSC membrane. Magenta, phagocyte cytosolic marker.

**Supplemental Movie S3. Test for TSC soma internalization of *scrm-1(tm698)* mutants.** Green, TSC membrane. Magenta, phagocyte cytosolic marker.

**Supplemental Movie S4. Test for TSC soma internalization of *cps-6(ok1718)* mutants.** Green, TSC membrane. Magenta, phagocyte cytosolic marker.
